# Supplementary material for: Are carbon emissions trading and green financial instruments synergistic? -Comprehensive quantitative research based on content analysis
Source: PLoS One. 2024 Mar 7;19(3):e0298601. doi: 10.1371/journal.pone.0298601 (PMC10919601; doi:10.1371/journal.pone.0298601)
Supplement: S1 Data — (ZIP) [file pone.0298601.s001.zip › Data/地级市Z_F工作报告.zip!/─■╧─/╬Γ╓╥╩╨/└┤╫╘╣1⁄2╓┌║┼í╛┬φ┐╦╩2╛▌═°í┐_2011.docx]

各位代表：

现在，我代表市人民政府向大会作政府工作报告，请连同《吴忠市国民经济和社会发展第十二个五年规划及2020年远景目标纲要（草案）》一并审议，并请市政协委员和其他列席人员提出意见。

2010年工作及“十一五”简要回顾

刚刚过去的2010年，是“十一五”决战之年，也是新世纪头十年里极富挑战的一年。在自治区党委、政府和市委的正确领导下，我们坚持以科学发展观为统领，努力克服各种困难和矛盾，坚定信心，锐意进取，全面完成市三届人大三次会议确定的各项目标任务，全市改革开放和现代化建设取得新的成就。

——经济运行稳中趋快。预计全年完成地区生产总值210亿元，增长13%。三次产业分别增长8.2%、15.6%和11%。地方财政收入31.5亿元，增长78.9%，其中一般预算收入14.9亿元，增长20.5%。全市社会消费品零售总额预计实现52亿元，增长15%。物价上涨得到有效遏制。

——发展活力不断增强。完成全社会固定资产投资216亿元，增长30%。金融机构存款余额269.7亿元，贷款余额243.5亿元，分别增长56.3亿元和32.8亿元。

——人民生活持续改善。城镇居民人均可支配收入13848元，增长9.5%。农民人均纯收入突破5000元关口，达到5041元，增长14.8%。城镇新增就业1.48万人。农村劳动力转移就业24万人，实现劳务收入14.4亿元。

——节能减排有效落实。全年万元GDP能耗下降8%，主要污染物二氧化硫和化学需氧量排放量分别减少3.4万吨和2400吨，全面完成“十一五”节能减排任务。

——重大活动圆满成功。成功举办2010宁夏“黄河金岸”国际马拉松赛和吴忠建城2224年系列纪念活动，地方形象得到全面展示和新的提升。

这些成就，是在经历各种严峻复杂形势下取得的，全市上下付出了极大努力，做了大量艰苦细致和卓有成效的工作。

（一）坚持项目带动，“两大任务”再获突破。牢固树立机遇意识，把“两大任务”作为第一要事，全方位多渠道开展上争项目、招商引资活动。全市共落实中央及自治区各类项目1965个，实际到位资金46.8亿元。实施招商引资项目351个，实际到位资金突破200亿元大关，完成目标任务的134%，是实施项目最多、完成投资最大的一年。成功引进浙江运达风机设备、山东新汶矿业煤机设备两家装备制造项目，填补了行业空白。引进中航郑飞粮食全价值产业链、伊利液态奶两家农副产品加工项目，为促进产业结构升级注入新的活力。

（二）加大协调服务，工业经济平稳运行。面对后金融危机影响和“十一五”节能减排攻坚的双重压力，及时出台进一步加快工业经济发展意见，果断采取资金帮扶、融资贴息等有效举措，助推工业经济优化升级、提速发展。预计全市规模以上工业企业完成增加值79亿元，增长16%。实施投资千万元以上的重点工业项目87个，完成投资103亿元。太阳镁业3.5万吨镁合金等45个项目建成投产，新增工业产值17亿元。清洁能源迅速发展，全市风电、光电装机总容量分别达到35万千瓦和3.5万千瓦。加大石化、冶金、建材等传统产业技改力度，实施技改项目54个。工业园区平台作用明显，基础设施不断完善，新入园企业67家，园区完成增加值占全部工业的80%以上。起步建设利通区毛纺织工业园，首批引进企业20家。针对严峻的节能减排形势，对电石、铁合金等高耗能企业实施限产、停产措施，加快11个重点减排工程建设，在有效控制能耗增长的同时，保持了工业经济稳定运行。

（三）注重提质增效，农村经济稳步发展。预计全年完成农业总产值65亿元，增长8.3%。全市优质粮食种植面积108万亩，粮食总产达8.8亿公斤，创历史新高。全市奶牛存栏17万头，增长13.3%。肉牛和羊饲养量分别达到59万头和552万只，分别增长24.2%和10.2%。设施农业、酿酒葡萄、红枣、高酸苹果、优质饲草、人工甘草种植规模不断扩大。孙家滩国家级农业科技示范园区建设全面启动。全市8个种养基地列入自治区现代农业示范基地范围，30个农产品获得无公害产地认定，233个获得产品认证，均居全区首位。新培育市级龙头企业22家，农民专业合作社达到65个，流转土地35.4万亩。盐池滩羊荣获中国驰名商标，天予圆枣、杞叶青饮品荣获全国农产品金奖，郭家桥刘湾大青葡萄荣获国家地理标志保护产品。大力度开展农田水利基本建设，改善灌溉面积46万亩，治理水土流失418平方公里。在全区“黄河杯”竞赛中再创佳绩，连续第三年获得组织奖并实现“县县得奖”。

（四）强化特色培育，第三产业明显活跃。培育交易额亿元专业市场6家，5亿元专业市场3家。涝河桥市场、东郊市场被列为全国“双百市场工程”。销售各类“家电下乡”产品12.8万台（件），销售总额达到2.8亿元。食品加工产业发展加快，实现产值100.2亿元，增长18%。成功举办2010首届吴忠房·车文化节，预计完成房地产投资28亿元，增长37%。加快打造以黄河大峡谷为主的特色旅游业，青铜古镇一期、黄河圣坛等工程基本完工，水工博物馆、黄河楼等重点项目加速推进。青铜峡拦河大坝、宁夏移民博物馆被列为国家第二批红色旅游项目。全年共接待游客147万人次，实现旅游收入7.6亿元。

（五）加大投入力度，城乡一体化步伐加快。全市共安排城乡建设项目297个，总建筑面积400万平方米，完成投资72.5亿元。全力推进滨河新区崛起战略，实施黄河金岸重点建设项目38个。新月广场、教育园区一期等重点项目建成投入使用，两馆一中心主体工程全面完成，世纪大道等6条城市道路、吴忠黄河公路大桥、S101线三期拓宽改造建成通车，新建、改造一批供水、供电、供热等基础设施，滨河大道市区过境段实现全程亮化。大力实施旧城改造提升战略，城市东南部建设全面启动。县城和城镇建设明显加快，实施盐池、同心新区建设与旧城改造、红寺堡城区路网与城北生态综合整治等一批县城重点工程。高闸、叶升等10个特色村镇建设进展顺利。新建古城湾新村等“塞上农民新居”示范点13个，综合整治旧村庄75个，改造农村危房4581户，在全区“塞上农民新居”考核验收中名列第一。新建改建农村公路760公里。生态建设持续有力，全市共完成营造林35万亩，新增绿地1万亩，水面6000亩，完成黄河两岸景观绿化1万亩。城市绿地率、绿化覆盖率分别达到30.8%和36.2%。青铜峡市荣获“国家园林城市”和“全国绿化模范市”称号。

（六）着力改善民生，社会事业全面推进。坚持教育优先发展战略，撤并整合中小学校60所，实施投资3.2亿元、25万平方米校安工程，一批安全、整洁的中小学校落成投入使用。利通区、青铜峡市、盐池县基本普及高中阶段教育，红寺堡区教育强县工作顺利通过自治区验收。加强文化阵地建设，利通区、青铜峡市标准化农家书屋在全区率先实现行政村全覆盖。深化医药卫生体制改革，国家基本药物制度全面落实，基本公共卫生服务均等化扎实推进。实施吴忠市精神病防治中心、青铜峡市人民医院等12个医院建设项目，完成19所乡镇卫生院、社区卫生服务中心改扩建工程，城乡医疗卫生服务网络进一步完善。“少生快富”工程扎实推进。全年人口自然增长率控制在9.53‰以内。吴忠市被评为全国婚育新风进万家活动示范市。全面解决养老保险历史遗留问题，稳步扩大社会保险参保范围，新型农村养老保险制度实现全覆盖。城乡低保补助标准进一步提高，特困群众大病医疗救助限额统筹分别由城市5000元、农村3万元统一提高到5万元。坚持以创业带动就业，发放全民创业贷款1.68亿元，新培养了一大批小企业和小老板。市财政安排1000万元专项资金，承诺为民办理的9件实事全面完成。建设廉租房9.6万平方米，经济适用房30万平方米，劳务移民租赁房1.8万平方米。认真研究解决失地农民养老保险问题，及时兑现征地拆迁、安置过渡费。科学、迅速、有效应对特大暴雨灾害，市财政拿出500万元支援同心救灾，建新村受灾群众一期150户安置房提前完工入住。第六次人口普查工作进展顺利。安全生产、应急管理、食品药品安全和防震减灾不断加强，人防、档案、外事、统计、住房公积金、妇女儿童、残疾人事业等有了新的发展。国防动员、军民共建和双拥工作取得新成绩。

（七）坚持依法行政，民主法制和政府自身建设进一步加强。深入推进依法行政工作，市人民政府被自治区评为依法行政先进集体。顺利通过自治区“五五”普法考核验收，获得优秀等次。深入开展工程建设领域突出问题和“小金库”专项治理工作，加大政府投资项目审计监督力度，政府公信力和群众满意度不断提升。强化社会治安综合治理，加强信访工作，最大限度解决群众合理诉求，有效化解了一批矛盾纠纷和信访突出问题。涉法涉诉信访案件化解工作连续两年居五市第一。深化“平安吴忠”创建活动，依法打击各种刑事犯罪，人民群众的安全感进一步增强。深入开展“民族团结宣传教育年”活动，民族团结、宗教和顺局面得到进一步巩固。高度重视并自觉接受人大法律监督和政协民主监督。办理人大代表议案、意见和建议60件，政协委员提案285件，取得良好效果。

各位代表！

伴随着2010年的胜利结束，“十一五”画上了圆满句号。回顾波澜壮阔的“十一五”，承载了全市人民继往开来的美好憧憬，也记录了新吴忠在关键阶段的砥砺奋进和全力追赶。经历了“十五”时期两次重大行政区划调整，新的市情和发展新要求赋予了“十一五”更加特殊而艰巨的历史使命。我们顶住了总量下降、实力削弱等困难和压力，紧紧抓住国家实施西部大开发和支持宁夏加快发展的历史机遇，谋长远，打基础，再造新优势，办成了一系列大事，办好了一系列喜事，办妥了一系列难事，全市经济社会迈上了新台阶。这五年是综合经济实力显著增强的五年。全市地区生产总值年均增长12.4%，经济总量是2005年的2.1倍。地方财政一般预算收入年均增长23.9%，收入总量是2005年的2.8倍。全社会固定资产投资五年累计达到650亿元，是“十五”时期的2.9倍，年均增长25.5%。这五年是自我发展能力明显提升的五年。工业“两城五园”格局初步成型，农业“十大基地”初具规模，第三产业形成了以食品加工、商贸物流、旅游和房地产为支撑的新特色，三次产业之比调整为12.7：55.6：31.7，经济内生动力显著增强。全市粮食总产量实现连续8年增产。这五年是城乡面貌发生巨大变化的五年。建成了一批重要的交通、水利和公共基础设施，公路通车里程达5760公里，比2005年增加895公里。市区建成区面积由2005年的23平方公里扩展到58平方公里。生态建设成效显著，五年累计植树造林140万亩，森林覆盖率达到12%。吴忠市被评为中国优秀生态旅游城市。这五年是对外开放实现重大突破的五年。成功举办迎奥运火炬传递、庆祝地级吴忠市成立十周年庆典等大型活动，吴忠市被确定为全国中小学生书法节首创地，被授予“国际友好城市特别贡献奖”。首次争创即获得“全国双拥模范城”命名。五年累计招商引资到位资金500亿元。先后有12家国内500强、民营500强企业落户吴忠。这五年是人民群众得到更多实惠的五年。城镇居民人均可支配收入和农民人均纯收入年均分别增长13%和11.8%。五年新增城镇就业和累计转移农村劳动力分别达到6万人和90万人。基本实现了城乡养老、医疗保险制度全覆盖。五年减少绝对贫困人口16.5万人，累计建成移民新村21个，完成生态移民18万人，解决农村46万人安全饮水问题。这五年是县域经济活力不断增强的五年。不断丰富和完善统筹发展、分类指导的工作思路，坚持每年组织开展县域重点项目、生态建设、农田水利建设观摩评比，加大以奖代补力度，加快扩权强县改革进程，进一步调整市辖区行政管理职能，极大调动了县域经济发展积极性。盐池县财政一般预算收入突破2亿元，位居全区山区8县之首。青铜峡市跻身西部百强县第32位。

各位代表！

总结2010年，回顾“十一五”，成绩值得肯定，经验弥足珍贵。五年来，特别是市委三届四次全会以来，我们始终以科学发展观为统领，坚持思想解放不停滞，坚持改革创新不动摇，坚持狠抓落实不懈怠，坚持转变作风不放松，集中群众智慧，激发社会活力，凝聚各方力量，最大限度地调动一切积极因素，营造了争先进位、干事创业的浓厚氛围。干部群众的思想观念和精神面貌发生了深刻变化，阻碍科学发展的体制机制障碍不断革除，科学发展的理念更加深入人心，加快发展的积极性、主动性、创造性空前迸发，形成了聚精会神搞建设、一心一意谋发展的强大精神动力。

这些成绩的取得，来自于市委的坚强领导，来自于市人大、政协的依法监督和理解支持，来自于全市上下、社会各界的共同努力。在此，我代表市人民政府，向为吴忠发展付出辛勤劳动的全市各族人民，向给予政府工作大力支持的人大代表、政协委员、民主党派、工商联、人民团体、离退休老同志和社会各界人士，向为吴忠改革发展稳定做出积极贡献的政法干警、驻吴人民解放军、武警和消防官兵，向所有关心、支持吴忠发展的中央及区属驻吴单位，表示崇高的敬意和诚挚的感谢！

我们也必须看到，在前进道路上还存在不少困难和问题。主要是：经济总量小，发展基础弱，科技支撑和创新能力明显不足，增长质量和效益还不理想，综合竞争力有待进一步提高；工业主导作用发挥不明显，“两高一资”问题依然严重，节能减排、环境保护压力比较大，优化升级和结构转型任务艰巨；农业增长缓慢，产业化程度较低，大型龙头企业较少，带动能力弱，现代农业推动难度大；统筹城乡发展不够，现代服务业发展相对滞后，教育、卫生等社会事业欠账较多；政府自身建设与人民群众的要求还有较大距离。所有这些，我们将在今后的工作中切实加以解决，努力把各方面工作做得更好。

“十二五”时期的基本形势和要求

“十二五”时期，是全面建设小康社会的关键时期。我们必须正确判断宏观形势的发展变化，充分认识新形势下面临的机遇挑战，审时度势，顺时应势，牢牢掌握工作主动权。根据中央、自治区和市委的分析判断，今后五年，我市仍将处于重要战略机遇期，经济社会发展既面临难得的历史机遇，也存在新的压力和挑战。总体来看，国家西部大开发战略继续向纵深推进，国务院支持宁夏一系列政策的全面落实，东部地区产业加快转移，为我们加快发展提供了新的契机。国家确定的呼包银经济区、宁夏沿黄经济带、“金三角”能源化工基地等区域经济发展布局，加大对革命老区、民族地区、贫困地区的扶持力度，为我们加快发展提供了良好环境。自治区打造黄河金岸特色产业带、实施内陆开放型经济发展战略，为我们加快发展创造了更为广阔的空间。全市上下在“十一五”时期创造的物质条件、奠定的思想基础和形成的良好作风，将为今后五年实现又好又快发展助推加力。同时我们也必须清醒看到，未来五年的国内外经济环境将更趋复杂，宏观经济稳定增长存在诸多不确定因素。区域经济之间的竞争日益激烈，实现赶超的压力将会越来越大。我市经济社会发展中的一些深层矛盾和问题仍将不断显现，改革、发展、稳定面临新的考验。讲机遇难得，必须紧紧把握；有困难再多，必须全力攻克。只要我们始终保持警醒，珍惜机遇，抢抓机遇，用好机遇，始终居安思危，团结奋进，就一定能开创新局面，实现新跨越。

“十二五”时期经济社会发展的总体要求是：以中国特色社会主义理论为指导，深入贯彻落实科学发展观，以转变经济发展方式为主线，以提升综合实力为核心，以深化改革创新为动力，以项目建设为载体，深入实施西部大开发战略，更加注重以“两城五园”为龙头的新型工业化，更加注重以“十大基地”和“两大示范区”为主攻方向的农业现代化，更加注重以商贸流通和特色旅游为主要内容的现代服务业发展，更加注重以建设“滨河水韵吴忠”为核心的特色城市化，更加注重以统筹城乡和山川协调发展为主要目标的基本公共服务均等化，更加注重以生态移民为重点的扶贫开发，强力推进全市经济社会持续快速发展，为全面建成小康社会打下更加坚实的基础。

必须在提升综合实力上实现重大突破。解决一切问题的关键在于发展，不论形势如何变化，加快发展始终是我们的第一要务。综合分析各种因素，“十二五”我市经济增长必须保持在年均13%以上的增速，到2015年全市地区生产总值在“十一五”末的基础上翻一番，达到420亿元。人均地区生产总值达到28900元（约合4000美元），力争接近全区平均水平。只有这样，才能确保到2020年与全国同步进入全面小康社会。全市地方财政一般预算收入达到37亿元，年均增长20%以上。

必须在转变发展方式上实现重大突破。始终把加快转变发展方式贯穿始终，深入实施工业强市战略，加快培育壮大“四大优势产业”，改造提升传统产业，使工业增加值实现翻番，突破200亿元，年均增长16%。加快发展现代农业，依托“十大基地”，培育壮大“五大支柱产业”，使优势特色农业占农业总产值的比重稳定在85%以上。加快发展现代服务业，按照产业化、高端化要求，培育做大现代服务业，使服务业增加值超过150亿元，从业人员比重达到40%左右。三次产业结构调整为8：57：35，科技对经济增长贡献率达到50%以上。

必须在统筹城乡发展上实现重大突破。以健全城市基础设施、优化城市发展环境、提高城市文明程度为抓手，创建文明城市；以打造滨河生态水韵之城、建设沿黄城市带核心区为抓手，创建生态城市；以加强社会治安综合治理和防灾减灾应急体系建设为抓手，创建平安城市；以改善城市生产和生活条件、增加就业机会、提高居民收入、完善社会保障体系为抓手，创建幸福城市。坚持统筹城乡发展的原则，实施“六到农家”工程，农村安全饮水普及率达到90%以上，新建和改造农村公路3000公里，全市城镇化率提高到50%以上。

必须在生态建设和环境保护上实现重大突破。继续实施退耕还林、退牧还草和三北防护林（五期）、天然林保护工程，加大力度保护好哈巴湖、罗山国家级自然保护区、青铜峡鸟岛自治区级湿地，推进防沙治沙示范区建设。到2015年，全市森林覆盖率达到15%，草原植被覆盖率达到80%，城市建成区绿化覆盖率达到40%，城市集中供热普及率达到95%以上，生活垃圾无害化和污水处理率继续提高，环境治理取得明显进展。与2010年相比，单位GDP能耗降低12%、主要污染物排放总量降低12%，资源节约型和环境友好型社会建设取得新进展。

必须在对外开放扩大投资上实现重大突破。主动参与国内外区域合作，开展形式多样的对外交流，不断拓展国际国内两大市场，实现面向周边地区，面向中东部地区，面向世界的全方位开放，打造内陆开放型经济示范区。上争项目、招商引资“两大任务”平均增幅保持在30%以上，全社会固定资产投资五年累计超过2500亿元，是“十一五”的4倍，年均增长30%。

必须在保障和改善民生上实现重大突破。始终坚持“强市”与“富民”同步的发展理念，努力保持每年公共财政投入80%以上用于民生事业，使人均基本公共服务接近全国平均水平。以人人享有生活保障为目标，力争城镇职工医疗保险、城镇居民医疗保险和新农合参保率均达到95%以上。城镇职工养老保险覆盖率达到95%以上，城市和农村居民养老保险覆盖率分别达到90%以上。城镇低保水平达到每人每月500元，农村达到300元。五年城镇新增就业人口8万人，转移农村劳动力100万人。完成7万人生态移民搬迁，解决26万贫困人口的脱贫问题。到2015年，城乡居民收入分别达到24500元和8900元，年均分别增长12%。人口自然增长率控制在10‰以内。

必须在维护社会和谐稳定上实现重大突破。始终坚持把维护社会稳定作为构建社会主义和谐社会的首要任务，把维护民族团结稳定作为重中之重，努力争创国家级民族团结先进市。加强社会治安综合治理，群众对社会治安状况满意率达到90%以上。完善矛盾纠纷排查调处机制，确保信访案件处理率和信访群众满意率均达到95%以上。建立健全各项安全生产监管制度，确保到“十二五”末，每亿元GDP安全生产事故死亡人数在2010年的基数上下降10%。

2011年主要工作

2011年是全面实施“十二五”规划的开局之年。根据市委三届八次全体（扩大）会议精神，政府工作必须继续把保持经济平稳较快发展作为首要任务，以项目为抓手，以投资为突破，加快产业优化升级，加快黄河金岸建设，加快生态移民，着力统筹城乡发展，着力加强社会建设和改善民生，努力为全面实现“十二五”目标迈出坚实一步。

今年全市经济社会发展主要预期目标和约束性指标是：地区生产总值增长13%；地方财政一般预算收入增长20%；全社会固定资产投资增长30%以上；社会消费品零售总额增长20%；城镇居民人均可支配收入增长10%以上；农民人均纯收入增长10%；人口自然增长率控制在10.5‰以内，城镇登记失业率控制在4.3%以内。完成自治区下达的年度节能减排任务。

（一）突出“两大任务”拉动，保持投资对经济增长的强劲拉力。大项目带动大投资，大投资推进大发展。把“两大任务”作为扩大投资的主抓手，坚持任务不减、措施更硬，确保完成上争资金60亿元，招商引资到位资金260亿元，各增长30%以上。抢抓机遇争投资。紧紧依托国家西部大开发等战略机遇，紧盯国家、自治区投资方向和重点，全力以赴争取支持，落实一批基础设施、生态环境、民生事业、农业产业化、能源交通等方面的项目，确保项目建设和投资长期稳定增长。加快建设促投资。全力推进确定的100个重点项目建设。重点抓好吴忠热电厂等产业项目，南环路及南片区道路等基础设施项目，黄河文化体育会展中心、南片区安居工程等社会民生项目，加快青铜峡火车站改扩建、吴忠市城西污水处理厂等项目开工建设进度，尽快形成投资拉动效应。加大招商引投资。进一步创新招商引资方式方法，建立重大签约项目跟踪落实机制，在取得实效上下功夫。力促中冶40万吨石头造纸、通达煤化工循环经济产业园开工建设。积极探索联办工业园等合作模式，加大承接东南沿海经济发达地区产业转移力度。重点围绕新能源、新材料、食品加工、装备制造、毛纺织、农副产品深加工等产业，加快引进技术水平高、带动力强的大企业和大项目，优化投资结构，推进产业升级。

（二）突出“两城五园”支撑，加快推进新型工业化进程。按照“园区化承载、集群化发展”的思路，着力调结构、转方式，扩总量、提效益，确保规模以上工业增加值增长16%。全力抓好重点项目。实施投资千万元以上重点工业项目100个，力促宁夏运达400台（套）风机制造等58个新项目开工建设。切实加快伊利年产19万吨液态奶等42个续建项目建设步伐。全力确保太阳镁业3.5万吨镁合金等45个建成项目达产达效，力争年内新建成项目50个，完成工业投资135亿元，增长31%。发展壮大新兴产业。集中力量实施新能源、新材料、煤及煤化工三大战略新兴产业培育工程，力争到年底全市风电、光电总装机容量达到80万千瓦，镁及镁合金产能达到15万吨，煤炭开采能力达到500万吨。全力实施装备制造等优势特色产业提升工程，着力发展以银星能源为龙头的风光发电装备制造业，以吴忠仪表为龙头的精密仪器仪表制造业，使装备制造业成为我市发展新兴产业的重要支撑。启动新一轮亿元企业成长工程，紧紧围绕技术创新和产品更新换代，培育发展大企业群体，确保年内新增亿元企业16家。大力扶持中小企业。高度重视中小企业的培育和发展，进一步完善扶持中小企业发展的政策措施，建立中小企业项目审批“绿色通道”。切实发挥各类担保平台作用，帮助中小企业融资做大，努力培育一大批各具优势、富有特色的中小企业群体，使之成为支撑县域经济、吸纳就业的生力军。加强工业园区建设。继续把工业园区作为支撑工业发展的主阵地，进一步提升各园区服务、承载和招商能力，不断提高园区产业集中度和规模化水平，力争“两城五园”完成项目投资突破100亿元。鼓励和支持各县（市、区）打造特色园区，加快利通区毛纺织工业园、盐池青山石膏城等县域工业园区建设，规划建设宁夏慈善工业园。加快发展循环经济。实施大唐和大坝燃煤机组脱氮、富荣化工废水治理、同盛化工尾气余热利用等10项重点节能项目和减排工程。严格新建项目环评管理，坚决管住新增排量。加大重点企业节能减排监管力度，下决心淘汰一批落后产能，确保实现年度节能减排目标。

（三）突出“两大示范区”引领，增创农业发展新优势。坚持农业基础地位不动摇，大力发展现代农业，扎实推进农业“十大基地”提质增效，力争农业总产值达到68亿元，增长5%。做大产业基地。全市粮食总产量稳定在8.5亿公斤以上。新增奶牛3万头，肉牛5万头，滩羊20万只，设施农业1万亩，红枣6万亩，葡萄3.6万亩，人工甘草10万亩，饲草20万亩。重点建好利通区冬麦及复种等规模种植基地和同心肉牛等规模养殖基地。全面加快孙家滩国家级现代农业科技示范园区建设，紧紧围绕设施农业、奶牛肉牛养殖、精品林果生产、生态观光四大产业发展，做足做好高效、节水、示范、生态等文章，使其成为具有较强辐射带动与示范效应的现代农业园区。做强龙头企业。积极引进支持各类农产品加工龙头企业重组扩张，稳步提升农业龙头企业市场竞争力和带动力。新培育或引进5家大型龙头企业，新增10个示范性专业合作组织。继续推进农产品市场建设，完善冷链保鲜、质量检测、物流配送等体系，健全营销网络，提高农产品流通现代化水平。积极开展农产品地理标志登记、认证认定和商标注册，打响“鲜果之乡”、“滩羊之乡”、“甘草之乡”、“圆枣之乡”、“葡萄之乡”等原产地品牌，着力培育一批宁夏著名、国内驰名品牌，加大宣传推介力度，使更多绿色、有机农产品打入区内外高端市场。夯实农业基础。继续坚持大规模高标准抓好农田水利基本建设，加强灌区农田渠系更新改造和山区高效节水设施建设。完成农田整治80万亩，改造中低产田8万亩，新增节水灌溉面积25万亩，发展高效节水补灌面积5万亩，治理水土流失面积240平方公里。解决10万人安全饮水问题。加强防汛抗旱工作，建立健全灾害防御体系，进一步提高农业抗灾避灾能力。提升科技水平。加快建立农业科技信息、科技创新、科技培训和科技服务体系，稳定科技特派员队伍。进一步加强与宁夏大学、陕西杨凌职业技术学院等区内外大专院校的科技合作，大力推广应用新技术、新产品，加快农业科技成果转化应用，提高科技支撑能力。

（四）突出民族特色优势，加快发展现代服务业。坚持把推动现代服务业发展作为产业结构优化升级的战略重点，努力推动服务业向高端化领域进行“二次创业”。重振商贸流通业。启动滨河大道旅游休闲街建设，推动老城商贸核心区提档升级，着力打造精品商贸区。加快十大市场、三大汽车服务区建设，积极推进红寺堡综合市场、青铜峡铁路物流园、盐池永生物流园、同心专业市场等县级市场建设，构建以利通区为中心的新型物流业发展格局，把我市建成宁夏乃至西部重要的物流集散地。深入推进“万村千乡、农超对接”等现代市场体系工程建设，继续做好“家电下乡”工作。做响特色旅游业。大力推进青铜峡黄河大峡谷旅游区建设，加紧建设黄河楼等一批标志性文化旅游设施，提升景点设施的档次和水平。重点发展以黄河文化游、红色经典游、绿色生态游和周边休闲游为主的旅游业。举办“黄河金岸”文化旅游节，着力打造西部特色旅游目的地。力争全年接待游客和旅游收入分别增长17%和20%。稳定发展房地产业。认真落实国务院、自治区关于保持房地产业健康稳定发展的一系列政策措施，规范市场秩序，发挥房地产业对投资和消费的双向拉动作用，促进房地产市场平稳健康发展。

（五）突出黄河金岸建设，加快城乡一体化进程。抢抓自治区发展沿黄城市带机遇，进一步加快城乡统筹步伐，改善人居环境。全年实施城乡建设项目257个，概算投资168亿元，增长30%。大手笔推进黄河金岸建设。加快黄河两岸30平方公里区域开发建设，实施CBD中心、黄河文化展示园、美食购物广场、滨河五星级酒店、黄河吴忠城市过境段砌护及综合整治等30个重点项目。新建、续建龙河唐都、黄河外滩城市社区等38个房地产项目和利华街南段、经二街等城市道路。推动商贸、金融、医疗、通讯等居民生活必需的服务网点布局，吸引人流、物流、资金流向城市新区聚集，不断增强辐射聚集能力。全方位抓好县城改造提升。加快利通区城市东南部市场、道路、绿化、水系等基础设施建设，确保年内南环路和南一环路竣工通车。抓好红寺堡区城区道路、青铜峡市龙海花园三期、盐池县金融大厦、同心县新区开发等项目，进一步提升县城建设品位。紧抓国家实施“百县中心镇工程”建设机遇，大力推进村镇整合集聚，抓好邵刚、韦州、惠安堡等中心集镇建设。深入实施塞上农民新居、危窑危房改造，推进农村民居适度集中。开工建设利通区至红寺堡区快速通道。新建、改建农村公路760公里。多层次提升生态建设水平。全市营造林30万亩，新增城市绿化面积1.3万亩，新增城市湖泊湿地3000亩。实施黄河沿线绿化、黄河湿地恢复和保护等生态绿化工程，构筑贯通黄河两侧辐射2公里的生态防护林体系。加强城乡环境综合整治，实施生态环境恢复暨生活垃圾资源化治理和16个农村环境连片整治示范项目，创建一批环境优美乡镇、文明生态村。利通区、盐池县加快创建国家园林城市，红寺堡区、同心县加快创建自治区园林城市。

（六）突出改革开放带动，增强经济社会发展活力。改革是发展的动力，也是实现科学发展的重要保证。推进重点领域改革。巩固政府机构改革成果，进一步理顺市与市辖区、市与直管县的职责权限，充分调动积极性。完善规范招投标和政府投资项目代建制管理。鼓励引导民间资本进入资源开发、基础设施、公用事业等领域，支持非公有制经济加快发展。深化农村改革，规范和完善农村土地承包经营权流转，逐步扩大推广范围。严格规范农村宅基地建设管理，坚决杜绝乱占滥建行为。严格执行土地利用总体规划和年度利用计划，确保占补平衡。提高对外开放水平。坚持以开放促改革，扩大开放领域，加快开放步伐。在巩固与区内外友好城市经贸合作的同时，进一步扩大面向中东国家和地区的开放，建立内外联动、互利共赢的内陆开放经济体系，更好地参与区域经济大循环。高标准办好2011宁夏“黄河金岸”国际马拉松赛，进一步推介和展示吴忠。

（七）突出保障和改善民生，着力推进社会事业全面发展。民生连着民心，发展必先惠民。把关注民生、改善民生作为政府各项工作的出发点和落脚点，努力促进基本公共服务均等化。认真办好市政府承诺为民办理的实事。把关注民生的着力点放在实施生态移民上，重点在利通区、红寺堡区、青铜峡市及盐池县扬黄开发区规划建设16个移民安置区，完成4万山区贫困人口的移民搬迁。坚持生态移民与劳务移民、产业移民、教育移民相结合，加快贫困群众的脱贫步伐，改善贫困群众的生产生活条件。解决10万人安全饮水问题。把关注民生的着力点放在促进教育均衡发展上，加快学前教育规范化，小学教育城镇化，中学教育城市化进程，积极调整职业教育结构，加大学校布局调整力度，逐步满足人民群众对优质教育资源的需求。开展利通区教育均衡发展示范区创建工作。完成红寺堡区基本普及高中阶段教育和同心县创建教育强县目标，加快创建教育强市步伐。把关注民生的着力点放在提升医疗卫生服务上，扎实推进医药卫生体制改革，积极稳妥推行公立医院改革，进一步健全医疗卫生服务体系。吴忠市人民医院创建成三级医院。积极推进人口计生综合改革，加强流动人口计生服务与管理，提高出生人口素质。把关注民生的着力点放在繁荣文化体育事业上，大力开展全民健身运动，广泛开展广场文化活动。充分挖掘历史文化、黄河文化、红色文化内涵，创作花儿歌舞剧《曼苏尔》等精品剧目，加快文化产业发展。进一步加强文化市场监管，净化文化市场环境。把关注民生的着力点放在完善社会保障体系上，进一步扩大各项社会保险覆盖范围。逐步提高城乡低保和企业退休人员基本养老金标准。建立健全城乡困难群体、特殊群体、优抚群体社会保障机制，加快面向老年人、孤儿、残疾人等群体的福利基础设施建设，利通区实现“自治区无障碍城市”创建目标。建立健全失地农民社会保险制度，做好失地农民就业与生活保障工作。扎实推进医疗保险市级统筹，探索建立异地就医结算制度。把关注民生的着力点放在解决群众实际困难上，深化创业型城市创建工作，有效落实就业补贴、小额担保贷款、创业促就业等政策。新增就业1.3万人，培训农村劳动力30万人，转移就业22万人，实现劳务收入12亿元。建立以公共租赁房、廉租房、经济适用房等互为补充的住房保障体系，缓解群众住房困难。完成城市东南部120万平方米的安置楼工程，让为城市建设做出奉献的群众早日住上宽敞明亮的新房。高度关注通胀预期，严厉查处扰乱价格秩序的违法行为，稳定基本生活用品市场价格，切实保障好老百姓的“菜篮子”、“米袋子”。

（八）突出和谐稳定，巩固安定团结大局。稳定是发展的前提，是和谐的基础。进一步维护好民族团结稳定。紧紧围绕“共同团结奋斗、共同繁荣发展”的主题，深入开展民族团结教育活动，扎实推进民族团结进步事业模范单位创建活动，巩固和发展平等团结、互助和谐的社会主义新型民族关系。依法管理宗教事务，坚决打击非法宗教活动，确保宗教领域稳定。进一步推进“平安吴忠”建设。加强社会治安综合治理，继续推进社会治安防控体系建设。做好信访、人民调解工作，有效预防和妥善处置群体性事件。加强公安执法规范化建设，注重警务机制改革创新，全面提升公安工作整体水平。继续巩固并扩大禁毒人民战争成果，严厉打击各类毒品犯罪活动。加强消防安全建设，强化煤炭、化工、建筑以及特种设备、食品药品等重点行业的安全监管，坚决遏制重特大事故发生。健全自然灾害、突发事件的应急处理机制，提高公共安全保障能力。进一步加强精神文明建设。围绕创建文明城市，广泛开展文明单位、文明村镇、文明行业、和谐社区等精神文明创建活动。支持工会、共青团、妇联等人民团体参与社会管理。加强国防动员，推进双拥共建，再创“全国双拥模范城”。

（九）突出行政效能建设，提升政府自身建设水平。新的形势和新的任务，对政府工作提出了更高的要求。我们将进一步加强自身建设，提高行政效能，努力做到为人民服务，对人民负责，受人民监督，让人民满意。加强学习调研，增强创造力。深入开展学习型机关创建活动，进一步完善机关学习制度，广泛开展读书学习活动，引导各级干部注重学习借鉴，注重调查研究，注重思路创新，注重实践探索，进一步吃透上情，认清市情，了解下情，不断提升自身能力和工作水平。坚持依法行政，增强公信力。行政机关工作人员特别是领导干部要带头学法、守法、用法，依法办事，切实提高运用法治思维和法律手段解决经济社会发展中突出矛盾和问题的能力。各级行政机关要自觉在宪法和法律范围内活动，严格依照法定权限和程序行使权力、履行职责。自觉接受人大及其常委会的法律监督、政协的民主监督，善于接受群众和媒体监督，切实提高行政行为的公正性、合法性和公开透明性。提高行政效能，增强执行力。加快推进政府效能建设，建立市领导督办、重大项目跟踪服务机制，加大对重点工作、重点项目的协调和督查力度，对办事拖拉、执行不力、落实不实的责任单位和责任人，坚决问责；对失职渎职、滥用职权、吃拿卡要等行为严肃查处。大力营造坚决执行、立即执行的工作氛围，切实做到大事先办、要事快办。优化服务环境，增强吸引力。将创优投资环境摆上重要位置，进一步加大行政审批制度改革力度，减少审批环节，简化办事程序，大力推进“一站式”服务。在继续优化硬件环境的同时，下更大的功夫营造良好的政策环境、法制环境、市场环境和人文环境。坚持企业至上、客商至上、发展至上的服务理念，千方百计为投资者提供热情周到、细致入微的服务，自觉寓管理于服务之中，切实做到亲商、爱商、扶商，不断增强对外吸引力。加强廉政建设，增强凝聚力。全面落实廉政建设责任制，加强对关键岗位、关键环节和关键人员的监督制约，加强对规划审批、工程招投标、土地征用等领域的专项治理，严格控制会议、公车、公务接待等经费开支，严格控制各类检查、评比、庆典活动。加大各类违法违纪案件查处力度，坚决纠正损害群众利益的不正之风，切实做到节俭办事，廉洁干事，勤政为民。

各位代表！

新的征程已经开启，新的目标催人奋进。让我们高举中国特色社会主义伟大旗帜，紧密团结在以胡锦涛同志为总书记的党中央周围，在市委的坚强领导下，全面贯彻落实科学发展观，坚定信心，昂扬奋进，攻坚克难，扎实工作，继续为谱写全市人民美好生活新篇章而努力奋斗！
